# Pseudotyped AAV Vector-Mediated Gene Transfer in a Human Fetal Trachea Xenograft Model: Implications for *In Utero* Gene Therapy for Cystic Fibrosis

**DOI:** 10.1371/journal.pone.0043633

**Published:** 2012-08-24

**Authors:** Sundeep G. Keswani, Swathi Balaji, Louis Le, Alice Leung, Anna B. Katz, Foong-Yen Lim, Mounira Habli, Helen N. Jones, James M. Wilson, Timothy M. Crombleholme

**Affiliations:** 1 Center for Molecular Fetal Therapy, Division of Pediatric, General, Thoracic and Fetal Surgery, Cincinnati Children’s Hospital Medical Center and The University of Cincinnati College of Medicine, Cincinnati, Ohio, United States of America; 2 The Children’s Institute for Surgical Science, The Children’s Hospital of Philadelphia, Philadelphia, Pennsylvania, United States of America; 3 Gene Therapy Program, Department of Pathology and Laboratory Medicine, University of Pennsylvania School of Medicine, Philadelphia, Pennsylvania, United States of America; University of Pittsburgh, United States of America

## Abstract

**Background:**

Lung disease including airway infection and inflammation currently causes the majority of morbidities and mortalities associated with cystic fibrosis (CF), making the airway epithelium and the submucosal glands (SMG) novel target cells for gene therapy in CF. These target cells are relatively inaccessible to postnatal gene transfer limiting the success of gene therapy. Our previous work in a human-fetal trachea xenograft model suggests the potential benefit for treating CF *in utero*. In this study, we aim to validate adeno-associated virus serotype 2 (AAV2) gene transfer in a human fetal trachea xenograft model and to compare transduction efficiencies of pseudotyping AAV2 vectors in fetal xenografts and postnatal xenograft controls.

**Methodology/Principal Findings:**

Human fetal trachea or postnatal bronchus controls were xenografted onto immunocompromised SCID mice for a four-week engraftment period. After injection of AAV2/2, 2/1, 2/5, 2/7 or 2/8 with a LacZ reporter into both types of xenografts, we analyzed for transgene expression in the respiratory epithelium and SMGs. At 1 month, transduction by AAV2/2 and AAV2/8 in respiratory epithelium and SMG cells was significantly greater than that of AAV2/1, 2/5, and 2/7 in xenograft tracheas. Efficiency in SMG transduction was significantly greater in AAV2/8 than AAV2/2. At 3 months, AAV2/2 and AAV2/8 transgene expression was >99% of respiratory epithelium and SMG. At 1 month, transduction efficiency of AAV2/2 and AAV2/8 was significantly less in adult postnatal bronchial xenografts than in fetal tracheal xenografts.

**Conclusions/Significance:**

Based on the effectiveness of AAV vectors in SMG transduction, our findings suggest the potential utility of pseudotyped AAV vectors for treatment of cystic fibrosis. The human fetal trachea xenograft model may serve as an effective tool for further development of fetal gene therapy strategies for the *in utero* treatment of cystic fibrosis.

## Introduction

Cystic fibrosis (CF), which is the most common lethal monogenetic disease, results from the absence of a functional cystic fibrosis transmembrane conductance regulator (CFTR) protein [1]. A recently described CFTR−/− porcine model demonstrates a similar phenotype to that of a newborn human with CF. Abdominal lesions dominate the initial presentation, with meconium ileus, pancreatic destruction, early focal biliary cirrhosis, micro gall bladder, and abnormalities in bile ducts [2], [3]. These results suggest that CFTR is expressed in several organ systems and site-specific replacement of this single CFTR gene could potentially correct the deficiency, making gene therapy an attractive CF treatment modality.

At birth neither the human patients with CF, nor the new born CFTR−/− piglets, show any evidence of inflammation or morphological abnormalities in the airway or submucosal glands. However, with time the characteristic features of human CF including inflammation, infection, mucus accumulation, tissue remodeling, and airway obstruction manifest. Today, airway infection and inflammation, and associated lung diseases causes much of the morbidities and mortalities associated with CF [4], [5]. The airway epithelium and submucosal glands are appealing targets for gene therapy of pulmonary manifestations of CF because they express high levels of CFTR in the tracheobronchial tree (with relatively lower levels of expression found in the respiratory epithelial cells as compared to higher levels in the SMG), and they have been characterized as a potential location of airway stem cells [6]–[10].

Postnatal gene transfer of a functionally active CFTR gene has been limited by immunologic barriers to viral vectors [11]–[15]. Also, in the postnatal environment, mucus production and a relatively great distance of the submucosal glands from the trachea lumen have rendered gene therapy ineffective. In clinical trials, CFTR gene transfer was inefficient to either the surface epithelium or submucosal gland cells [16]–[19]. The discrepancy in gene transfer efficiencies between animal models and human clinical trials may be due to species-specific physiologic differences between humans and lower species [20]. Therefore, an improved model to study gene therapy in cystic fibrosis is necessary to better predict outcomes in clinical trials.

Considering the inefficiencies in postnatal gene transfer, we investigated alternative strategies for cystic fibrosis gene therapy. The fetus presents a potentially favorable environment for CFTR gene transfer including: 1) decreased physical barriers, 2) an immunologically permissive environment, 3) greater access to developing submucosal glands, and 4) the potential to transduce a respiratory-epithelial stem cell population. The development of a human fetal *ex vivo* model would provide the opportunity to screen potential gene transfer modalities in a species specific environment. We have previously reported a postnatal human bronchial xenograft model, where denuded rat tracheas repopulated with human bronchial cells are xenografted into nude mice, developed a fully differentiated pseudo stratified respiratory epithelium with occasional incompletely formed submucosal glands [21]. Applying a similar xenograft strategy to develop a human fetal *ex vivo* model, we implanted whole human fetal tracheas into a subcutaneous pocket in severe combined immuno-deficient (SCID) mice [22]–[24]. We have recently reported that this model recapitulates normal development of human fetal airway epithelium and tracheal SMG as per the staging system described by Thurlbeck *et al.* [25] after a 4-week engraftment period [24]. Using this xenograft model, we can examine viral-vector-cellular interactions and evaluate transduction efficiencies in a representative human fetal tracheal environment. Our earlier findings of efficient gene transfer in the fetal tracheal environment using adenoviral and pseudo-typed lentiviral-based vectors concurs with the suggestions of others that the fetal trachea may be a conducive environment for gene transfer [26]–[29].

Due to the limited duration of transgene expression with adenoviruses and the potential risk of insertional mutagenesis with lentiviruses, we chose to examine the effects of adeno-associated viral (AAV) vectors for gene transfer. These AAV vectors have a better safety profile, pose a lower risk for insertional events, and have potential for long term transgene expression [30]. In our previous study, utilizing an *in vitro* human fetal trachea model we reported efficient gene transfer using AAV2/2 to the fetal respiratory epithelium and submucosal glands [21]. Since the capsid is a major determinant of vector tropism, we hypothesized that a pseudotyping strategy, which replaces the capsid of the AAV 2 vector with capsid proteins from other AAV serotypes, could potentially enhance transduction efficiency. AAV serotypes 7 and 8 were isolated from non-human primates. These serotypes are thought to have sufficient homology to retain viral tropism for human target cells, but are divergent enough to avoid detection by pre-existing antibodies generated against commonly found human AAV serotypes [31], [32]. The pseudotyping strategy has demonstrated a unique transduction efficiency and tropism profile for each serotype in various tissues, including liver, muscle and skin [31], [33]–[35]. Despite these advantages, postnatal reports indicate that the pseudotyping strategy has not resulted in greatly improved gene transfer to the tracheo-bronchial tree [14], [36], [37].

In this study, we hypothesize that an AAV pseudotyping strategy in the fetal environment will result in enhanced gene transfer to the target cells of cystic fibrosis gene therapy. To test this hypothesis, using our validated human fetal trachea xenograft model, we compared the transduction efficiency of AAV2 and four AAV pseudotyped vectors (AAV2/1, AAV2/5, AAV2/7, AAV2/8) to the respiratory epithelium and submucosal glands. Further, to assess if it is the fetal environment that is permissive of enhanced gene transfer, we compared fetal transduction efficiencies to postnatal bronchial xenograft controls.

## Materials and Methods

### Ethics Statement

Human fetal tracheas were obtained from Advanced Bioscience Resources (Alameda, CA). Adult bronchial segments were obtained from The National Disease Research Interchange (Philadelphia, PA). The study protocol, which involves use of anonymous, de-identified, discarded human fetal or adult tissue, was reviewed and granted an exempt status by The Children’s Hospital of Philadelphia Institutional Review Board.

All animal procedures were approved by the Institutional Animal Care and Use Committee of The Children’s Hospital of Philadelphia. SCID mice were obtained from Charles River Laboratories (Wilmington, MA) ranging in ages 6–8 weeks. Mice were anesthetized with methoxyflurane inhalation for all procedures. After any procedure, mice were allowed to recover in incubators overnight and all efforts were made to minimize suffering.

### Pseudotyped Adeno-associated Virus Production

Pseudotyped adeno-associated vectors AAV2/2, 2/1, 2/5, 2/7 and 2/8 were obtained from the Vector Core at the University of Pennsylvania and produced as previously described [31]. The AAV2/2 serotype was constructed by standard transfection protocols and purified by single-step heparin chromatography. A pseudotyping strategy was used to produce AAV2 vectors packaged with the capsid proteins of AAV1, AAV5, AAV7 and AAV8. Briefly, recombinant AAV genomes equipped with AAV2 inverted terminal repeats (ITRs) were packaged by triple transfection of 293 cells with *cis*-plasmid, adenovirus helper plasmid, and a chimeric packaging construct. To create the chimeric packaging constructs, the XhoI site of p5E18 plasmid at 3,169 bp was ablated. The modified plasmid was then restricted with XbaI and XhoI in a complete digestion to remove the AAV2 cap gene and replace it with a 2,267-bp SpeI/XhoI fragment that contains the AAV1, AAV5, AAV7, or AAV8 cap gene. For all AAV vectors, the cDNA bacterial β-galactosidase was inserted as a reporter and driven by a CMV promoter. Pseudotyped recombinant vectors were purified by the standard CsCl_2_ sedimentation method. Genome copy (GC) titers of AAV vectors were determined by TaqMan (Applied Biosystems, Foster City, CA) analysis, using probes and primers targeting SV40 poly (A) region.

### Human Fetal Tracheas

The protocol, which involves use of anonymous, de-identified, discarded human fetal tissue, was reviewed and granted an exempt status by The Children’s Hospital of Philadelphia Institutional Review Board. Human fetal tracheas were obtained from Advanced Bioscience Resources (Alameda, CA) from 18 aborted fetuses ranging in age from 18 to 22 weeks based on prenatal ultrasound. The tracheas were placed in Dulbecco’s modified Eagle medium (DMEM, Gibco, Carlsbad, CA) containing 10% heat-inactivated fetal bovine serum (Gibco), penicillin 100 IU/mL, and streptomycin 0.1 mg/mL (Gibco). Cultures were incubated at 37°C for 24 hours to exclude infection before transplantation; no tracheas were lost to infection.

### SCID-human Fetal Trachea Xenograft Model

All animal procedures were approved by the Institutional Animal Care and Use Committee of The Children’s Hospital of Philadelphia. As previously described [24], SCID mice (Charles River Laboratories, Wilmington, MA) ages 6–8 weeks were anesthetized with methoxyflurane inhalation, and all efforts were made to minimize suffering. After a 5-mm incision was made in the right flank, a subcutaneous pouch was created superficial to the panniculus carnosus. Each fetal trachea (mean gestational age 20±0.4 weeks, range 18–22) was implanted into the pouch and secured by a single 8–0 Prolene suture (Ethicon, Somerville, NJ). The skin incision was closed with 5–0 Vicryl sutures (Ethicon) and covered with a transparent dressing (3 M Healthcare, St. Paul, MN).

### SCID-human Adult Bronchial Xenografts

The protocol, which involves use of anonymous, de-identified, discarded human tissue, was reviewed and granted an exempt status by The Children’s Hospital of Philadelphia Institutional Review Board. Human adult bronchial segments were obtained from the National Disease Research Interchange (Philadelphia, PA). Bronchial segments from a 35-year-old cadaver were sterilely dissected and placed in Dulbecco’s modified Eagle medium (Gibco) that contained 10% heat-inactivated fetal bovine serum (Gibco), penicillin 100 IU/mL, and streptomycin 0.1 mg/mL (Gibco). Bronchial segments were then incubated at 37°C for 24 hours to exclude infection before transplantation. After formation of the subcutaneous pockets in 9 SCID mice, the bronchi were placed in the pouch, enveloped by the panniculus carnosus, and secured with an 8–0 prolene (Ethicon). The skin was closed and a sterile transparent dressing was applied (3 M Healthcare). The bronchial segments were engrafted for 1 month.

### AAV Vector Delivery to Fetal Trachea and Adult Bronchial Xenografts

All 27 SCID mice tolerated implantation of fetal tracheas and adult bronchi. Four weeks after implantation, the tracheas and bronchi produced mucus, formed an operculum over the openings, and were well vascularized. To deliver vectors, SCID mice were anesthetized with inhalational methoxyflurane. A 1-cm incision was made in the skin at the caudal end of the xenograft. With exposure of the fetal trachea, mucus was extracted by insertion of a disposable microsyringe (Becton Dickinson, Franklin Lakes, NJ) through the operculum of the xenograft. Animals received 1×10^11^genome copies of AAV-CMV-LacZ, 2/1, 2/2, 2/5, 2/7, 2/8 vectors in 20 µL of phosphate buffered saline (PBS) (*n* = 3/vector) or PBS alone for controls (*n* = 3). The incision was closed and a sterile dressing was applied. One month after vector delivery, trachea xenografts were biopsied or harvested; 2 months later, animals were humanely killed and xenografts were removed for analysis. Similarly in the adult bronchial xenografts at 4 weeks, all specimens produced mucus, which was then extracted by microsyringe (Becton Dickinson) through a superficial incision. 1×10^11^ genome copies of the pseudotyped AAV 2/8 (*n* = 3) or AAV2/2 (*n* = 3) vectors were injected in a total of 20 µL of PBS or PBS alone for 3 controls. One month after vector delivery, adult bronchial xenografts were harvested and transgene expression was quantified in the respiratory epithelium and submucosal gland cells.

### Quantification of Transduction

Tracheas or bronchi were cut transversely into 2- to 3-mm pieces, washed in PBS, fixed in 0.5% glutaraldehyde for 15 minutes, and X-gal stained with a solution containing 1 mg/mL of 5-bromo-4-chloro-3-indoyl-β-D-galactopyronidase, 5 mmol/L K_3_Fe(CN)_6_, 5 mmol/L K_4_Fe(CN)_6_, and 1 mmol/L MgCl_2_ in PBS, pH 7.4. After overnight incubation at 37°C, samples were post-fixed with 10% neutral buffered formalin (Sigma) for 16 hours. Post-fixed X-gal stained tissues were mechanically processed and paraffin embedded. The 5-µm sections obtained were counterstained with 0.5% nuclear fast red. Images were obtained using a Leica microscope and analyzed with computer-assisted image analysis (Scanalytics, Fairfax, VA). A minimum of 9 sections per animal were analyzed for LacZ expression signified by cell specific green staining. Transduction efficiencies were quantified as the percent of positively stained respiratory epithelium and submucosal gland cells.

### Immunohistochemistry for AAV2/2 and AAV2/8 Receptors

The 5-µm paraffin sections from 18-week-old fetal tracheas and trachea sections from an adult cadaver (NDRI, Philadelphia, PA) were rehydrated in distilled water, immersed in Tissue Unmasking Fluid, pH 6.2 (Signet Laboratories, Dedham, MA), and microwaved (Ted Pella, Redding, CA) on high for 5 min to facilitate antigen retrieval. Slides were washed with distilled water and transferred to PBS. Samples were blocked using normal 10% goat serum in PBS (30 min at room temperature). Slides were then incubated with a mouse anti-heparan sulfate proteoglycan monoclonal antibody (1∶20 dilution, Chemicon, Temecula, CA) with normal 10% rabbit serum (for AAV2/2) or mouse monoclonal antibody for 67-kDa laminin receptor (1∶20 dilution, Abcam, Cambridge, MA) with normal 10% rabbit serum (for AAV2/8) for 30 minutes at room temperature and then overnight at 4°C. After slides were washed with PBS containing 0.1% triton, they were incubated for 30 min at room temperature with biotinylated species-specific IgG (1∶200, Vector Labs, Burlingame, CA) for AAV2/2 slides and Alexafluor 488 species-specific IgM (1∶200, Invitrogen, Carlsbad, CA) for AAV2/8 slides. The AAV2/2 slides were washed with PBS and avidin-biotin complex (Vector Labs) added. The slides were then rinsed in PBS, developed with chromagen 3, 3′-diaminobenzidine (Sigma), lightly stained with hematoxylin, and mounted with xylene-based mounting media. The AAV2/8 slides were washed with PBS and mounted using prolong gold media containing DAPI (Invitrogen).

### Statistical Analysis

Paired Student’s *t*-test and analysis of variance (ANOVA) were used to compare transduction efficiencies between the five AAV vectors. A *p* value of <0.05 was considered significant. Data were expressed as mean ± standard error of mean (SEM).

## Results

### Transduction Efficiency of AAV Pseudotypes in the Xenografted Human Fetal Trachea Model

**Figure 1:**
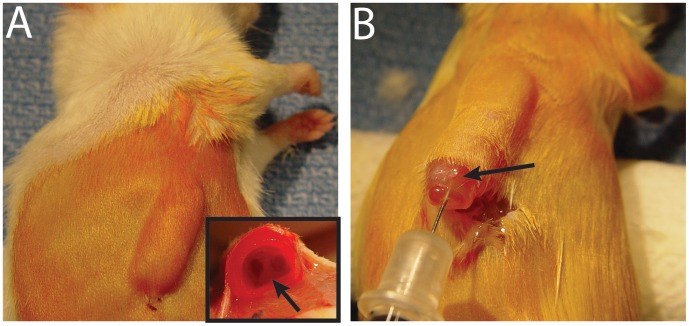
Human fetal trachea xenografts: Human fetal trachea xenografts 4 weeks after subcutaneous implantation in the SCID mice. A) Fetal trachea xenograft model. Inset demonstrates the biopsied cross sectional view of the xenograft with visible carina (arrow) at four weeks after subcutaneous implantation in a SCID mouse. (B) At four weeks after implantation, an operculum is formed over the trachea opening; xenografts produce mucous and are well vascularized (arrow). A microsyringe is inserted through the operculum to extract mucus and inject AAV vectors into the lumen of the xenografts.

In evaluating the effect of pseudotyping AAV2 vectors on transduction efficiencies in this model of implanted human fetal tracheas into subcutaneous pockets, all SCID mice tolerated implantation. At four weeks, the tracheas were well vascularized, formed operculum, and produced secretions (Figure 1). 1×10^11^ genome copies of AAV2/2 or pseudotyped vectors AAV2/1, 2/5, 2/7, and 2/8 were individually injected into the lumen of engrafted human fetal tracheas. PBS was injected in control xenografts.

**Figure 2:**
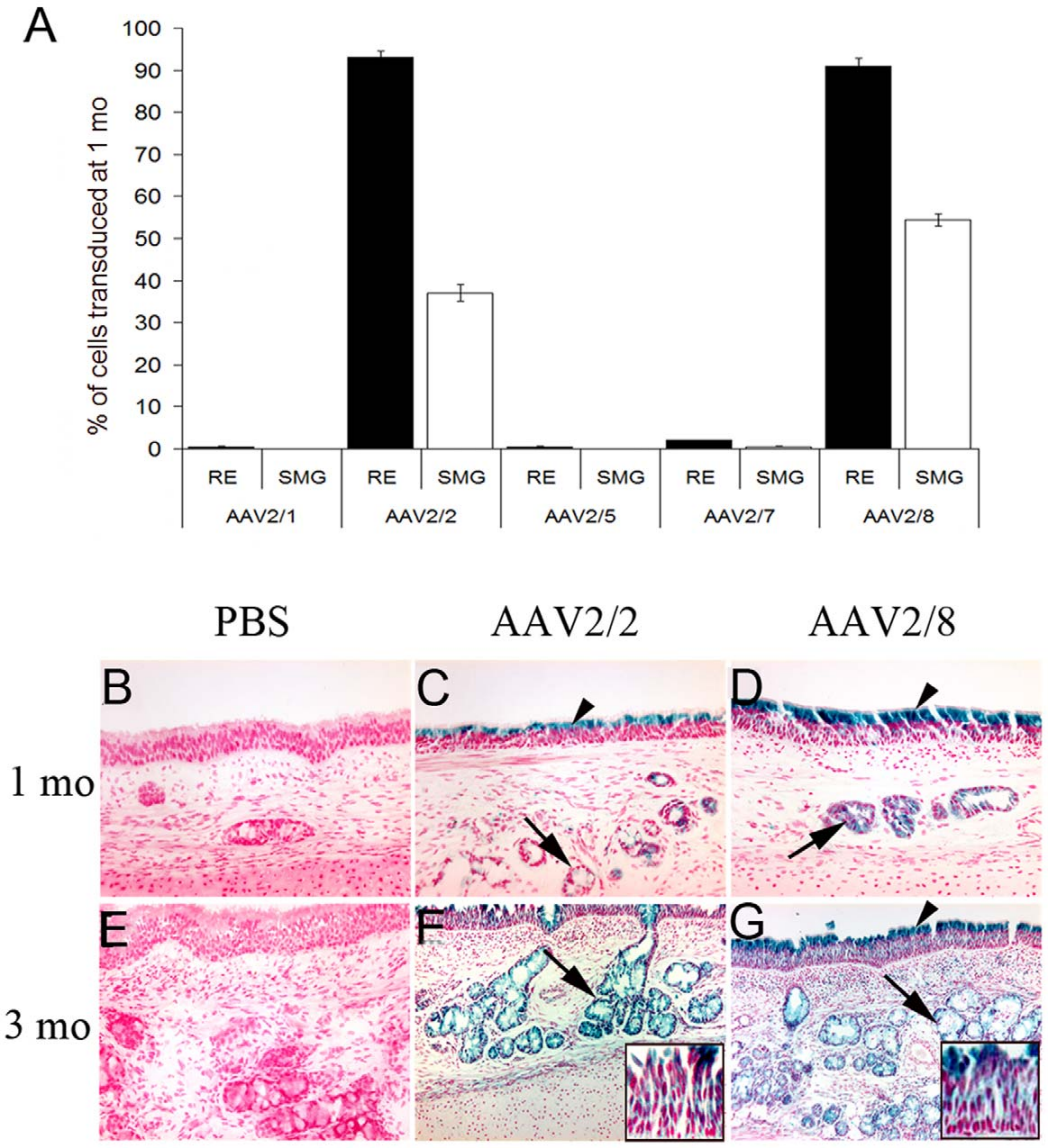
Transduction efficiencies of AAV2 and pseudotyped AAV2 vectors injected in fetal trachea. (A) Graph depicts percent of cell transduction efficiencies at 1 month by AAV2/2 and four pseudotyped AAV2 vectors (AAV2/1, AAV2/5, AAV2/7, AAV2/8) for respiratory epithelium (RE) and submucosal glands (SMGs). Bar plots represent average ± SEM. No LacZ positive cells were found in PBS control xenografts at 1 or 3 months (B, E). Only AAV2/2 (C) and AAV2/8 (D) demonstrate efficient gene transfer to the RE and SMG at 1 month. In comparison, other pseudotyped vectors AAV2/1, AAV2/5 and AAV2/7 minimally transduce the RE and SMG. By 3 months, there is near complete transduction to the RE and SMG by AAV2/2 (F) and AAV 2/8 (G). SMGs (arrow) and RE (arrowheads).

Control xenografts showed no positive LacZ staining at one month after PBS administration. Tracheal biopsies, one month after vector administration demonstrated that AAV2/2 transduced 93±1.5% of the respiratory epithelium and 37±2% of the submucosal gland cells. In xenografts injected with AAV2/1, 2/5 and 2/7, there was minimal LacZ expression in the tracheal biopsies; this finding was confirmed by further analysis on the entire xenograft. At 1 month, both AAV2/1 and AAV2/5 transduced <0.1% of the respiratory epithelium and 0% of the submucosal gland cells, whereas AV2/7 transduced <0.1% of the respiratory epithelium and 2.0±0.1% of the submucosal gland cells. In contrast with the other pseudotyped AAV vectors at 1 month, AAV2/8 transduced 91±1.9% of the respiratory epithelium and 54.4±1.4% of the submucosal gland cells. Specifically, AAV2/8 was significantly more efficient than AAV2/2 in transducing submucosal gland cells (54.4±1.4% vs. 37±2%, respectively) (p<0.005). With successful transduction observed only in serotypes AAV2/2 and 2/8, only these xenografts were maintained and harvested for subsequent evaluation. At 3 months, AAV2/2 and 2/8 demonstrated transgene expression >99% in both the respiratory epithelium and submucosal gland cells (Figure 2).

### Comparison of Gene Transfer Efficiency in the Adult vs. Fetal Respiratory Epithelium and Submucosal Gland Cells

**Figure 3:**
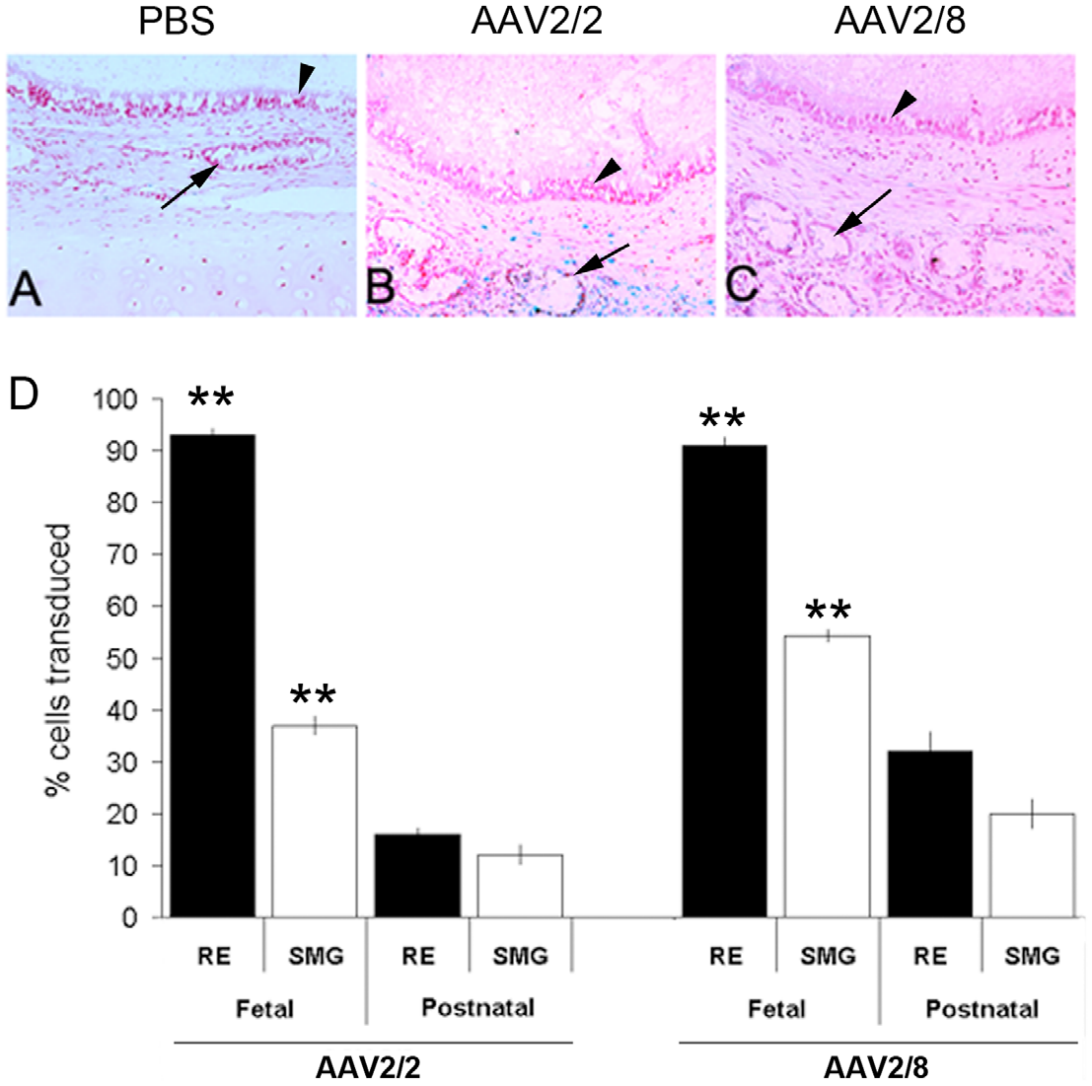
Transduction efficiencies of AAV2/2 and AAV2/8 in adult tracheobronchial xenografts. Transduction efficiencies of AAV2/2 and AAV2/8 were tested in an adult human bronchial xenograft. There was no LacZ positive staining in the PBS control adult xenograft (A). At 1 month, gene transfer to the adult postnatal xenograft with AAV2/2 (B) and AAV 2/8 (C) was minimal to the respiratory epithelium (RE) and submucosal glands (SMG). Arrows indicate SMG and arrowheads point to the RE. (D) Reduced cell transduction efficiency in postnatal xenografts compared to the human fetal trachea xenografts is demonstrated in the bar plot (average±SEM).

To confirm that the transduction observed with AAV2/2 and the pseudotyped AAV2/8 was unique to the fetal environment, we developed a postnatal xenograft control by implanting adult bronchial segments into subcutaneous pockets in SCID mice. After a 1 month engraftment period, 1×10^11^ genome copies of AAV2/2 or 2/8 were injected into the bronchial segments. PBS was injected in control xenografts. Transduction efficiency for the respiratory epithelium and submucosal gland cells for AAV2/2 were 16±1.5% and 12±2.1%, respectively. Gene transfer efficiencies for AAV2/8 to the respiratory epithelium and submucosal gland cells were 32±4.1% and 20±1.9%, respectively. PBS controls did not have any LacZ positive staining. Gene transfer with AAV2/8 was significantly greater than with AAV2/2 in both adult respiratory epithelium (32±4.1% vs 16±1.5%; p<0.01) and adult submucosal gland cells (20±1.9% vs. 12±2.1%; p<0.01) (Figure 3). Transduction by both AAV2/2 and 2/8 were significantly lower in adult bronchial xenografts compared with that observed in the fetal tracheal xenograft in both the respiratory epithelium and submucosal glands. Specifically, in respiratory epithelium, AAV2/2 was 16±1.5% in adult vs. 93±1.5% in fetal (p<0.0001), whereas AAV2/8 was 32±4.1% in adult vs. 91±1.9% in fetal (p<0.0001). In submucosal gland cells, AAV2/2 was 12±2.1% in adult vs. 37±2% in fetal (p<0.0001) and AAV2/8 was 20±1.9% in adult vs. 91±1.9% in fetal (p<0.0001) (Figure 3B).

### Comparison of AAV2/2 and AAV2/8 Receptors in Adult and Fetal Tracheas

**Figure 4:**
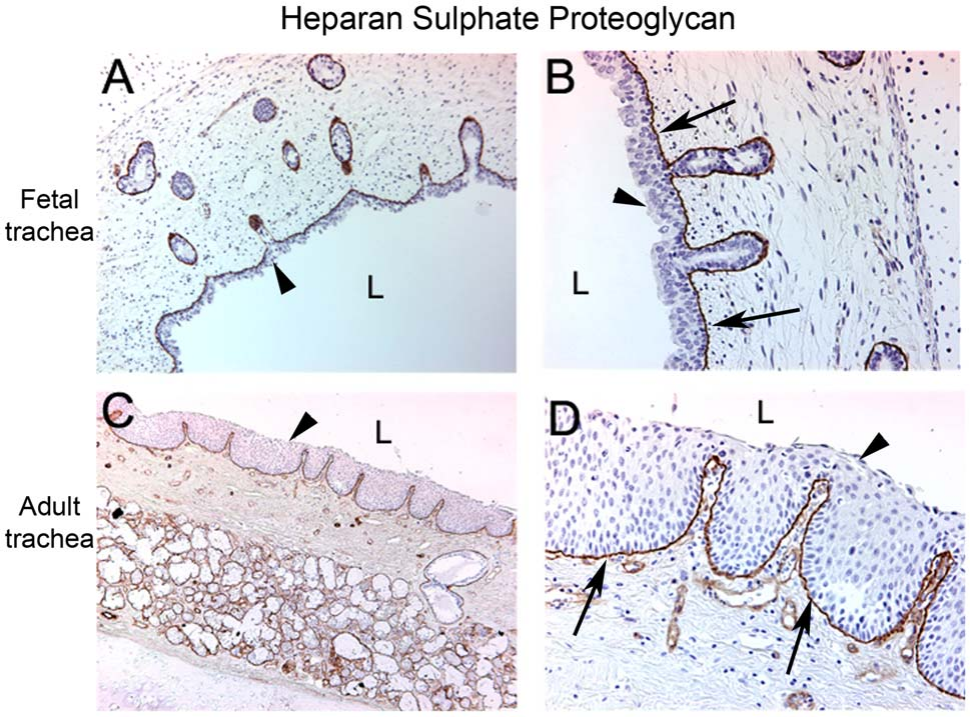
Immunostaining with heparan sulfate proteoglycan (HSPG), the AAV2 receptor on fetal and adult tracheas. Immunostaining at 10× and 40× magnification in fetal (A and B) and adult trachea (C and D) respectively. Expression of HSPG was limited to the basolateral surface of the respiratory epithelium (arrows). There is no staining of the airway epithelium (arrow heads) in either model. Luminal surface of the trachea (L).

We hypothesized that the significantly enhanced transduction efficiencies observed in the fetal trachea compared to postnatal controls could be due to accessibility of the viral receptors in the fetal trachea. To test this hypothesis, we compared immunostaining patterns between fetal and adult trachea samples for the AAV2 receptor, heparan sulfate proteoglycan (HSPG). Similarly, we performed immuno-histochemistry for a potential AAV2/8 receptor (67 kDa laminin receptor) [38]. Immunostaining in the adult trachea demonstrated expression of HSPG limited to the basolateral surface of the respiratory epithelium. In the fetal trachea, there was a similar basolateral distribution of HSPG expression. Although HSPG distribution was similar in adult and fetal tracheas, fetal submucosal glands are closer to the tracheal lumen and there is a decreased thickness of the fetal respiratory epithelium (Figure 4).

**Figure 5:**
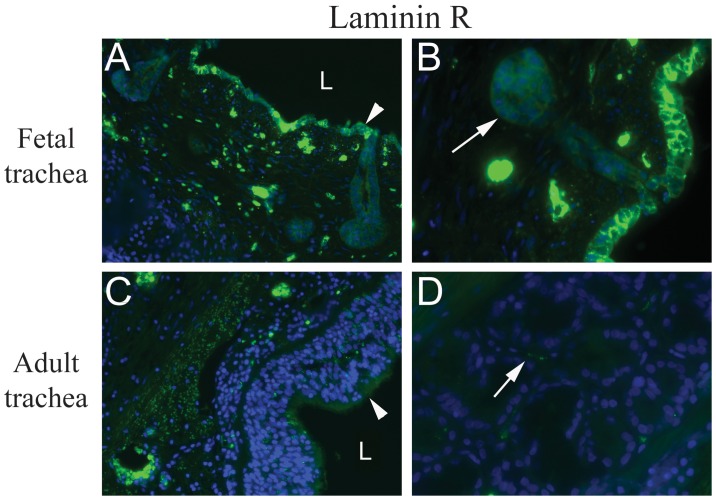
Laminin Receptor (R) staining of fetal and adult tracheas. Immuno fluorescent staining for Laminin R, one of the potential AAV8 receptors on fetal (A and B) and adult tracheas (C and D) at magnification 20× and 40×, repectively, demonstrates expression was most prominent in the surface epithelium (A, arrowheads) and submucosal glands (B, arrows) of the fetal trachea but minimal in the surface epithelium (C) and submucosal glands (D) in the adult trachea. Luminal surface of the trachea (L).

Immunostaining of a proposed AAV2/8 receptor (laminin R) demonstrated enhanced expression in both the respiratory epithelium and submucosal glands in the fetal trachea compared to minimal expression observed in the adult trachea (Figure 5).

## Discussion

The purpose of this study is to determine if an AAV pseudotyping strategy in the fetal environment can result in enhanced gene transfer to the target cells of CF gene therapy compared to postnatal controls. Our data demonstrate that AAV serotype 2 and the pseudotyped AAV2/8 vector result in highly efficient gene transfer in the respiratory epithelium and the submucosal gland cells of the fetal trachea compared to postnatal controls in the xenograft model. In light of previous reports of the limited postnatal transduction efficiency and rapid cellular turnover exhibited by the respiratory epithelium, the long term transgene expression with AAV2/2 and AAV2/8 are all the more impressive. At 1 month, transduction of the submucosal gland cells in fetal trachea is more efficient with AAV2/8. However, at 3 months, both AAV2/2 and AAV2/8 achieve highly efficient and stable transduction of the respiratory epithelium and submucosal gland cells. The increased transduction efficiency of AAV2/8 at one month may be related to its previously reported ability to more readily traverse biological barriers. In contrast to AAV2/2 and AAV2/8, the administration of AAV2/1, AAV2/5, and AAV2/7 demonstrate relatively low transduction efficiency in this model compared to AAV2/2 and AAV2/8. In addition, the highly efficient transduction of the submucosal gland cells may have significant implications for the prospective utility of fetal gene therapy to correct cystic fibrosis. The pseudotyped AAV vectors used in this study express a LacZ reporter gene. These are necessary preliminary data to determine the most effective AAV vectors for targeted gene delivery, with the eventual goal of significantly attenuating CF airway manifestations. Goldman *et al.* [21] demonstrated similar levels of transgene expression in human postnatal bronchial xenografts when transduced with either adenoviral CFTR or LacZ vectors. Similarly, we and several other groups have established that genetic reconstitution of CFTR results in functional correction of CF pathology in multiple models of the disease. These studies have also demonstrated a clear relationship between the dose of virus administration, efficacy of gene transfer, and the detected functional improvement, which partly explains the limited clinical success of postnatal gene therapy. In this context, the goal of this study was not to determine the genetic reconstitution of CFTR using AAV vectors, but to determine if the fetal environment is more permissive of gene transfer, and to further determine if a pseudotyping strategy with AAV vectors may result in enhanced tropism and transduction efficiency profiles. Based on our results, we plan to administer CFTR cDNA with a flag protein sequence or a reporter gene using the AAV serotype 2 and pseudotyped AAV2/8 vectors, and determine the effects on CFTR genetic reconstitution in a human fetal trachea xenograft model.

Efficient gene transfer by AAV2/2 and AAV2/8 was similar to our observations with other viral vectors in the fetal environment, such as adenovirus [28] and lentivirus [26], [27]. Despite significant differences in their transduction mechanisms, the excellent gene transfer of the vectors suggests that the fetal trachea may be uniquely permissive to gene transfer strategies. In a similar experiment implanting adult human bronchial segments as a postnatal control, we verified that the efficient transduction observed with AAV2/2 and AAV2/8 was due to unique properties of the fetal trachea and not due to the immuno-compromised SCID xenograft model. We found relatively efficient gene transfer to adult bronchial xenografts compared with previously reported postnatal gene transfer to the tracheobronchial epithelium; this difference was more pronounced in the submucosal gland cells. These results suggest that the xenograft model may favor increased gene transfer efficiency. However, gene transfer with both AAV2/2 and AAV2/8 were significantly lower at 1 month in the postnatal control than in the fetal trachea. Therefore, some differences inherent to the fetal trachea must permit the high efficiency gene transfer with AAV2/2 and AAV2/8. Our findings are consistent with the findings by Tarantal and Lee *et al* [39], who used AAV pesudotyping strategy for fetal gene therapy. Using a fetal monkey model, it was demonstrated that when administered sufficiently early in gestation, fetal gene transfer using AAV pseudotyped vectors results in: 1) site specific long term transgene expression, and 2) offers the possibility of eliminating disease as well as the potential for immune responses to transgene products and/or components of the vector(s). Interestingly, their results also demonstrate that different pesudotyped vectors and different routes of vector administration results in different transduction efficiencies. In a different study, Clemens *et al* [40] studied AAV serotypes 1 and 2, and demonstrated that expression profile of AAV vectors (both levels of transgene being expressed, as well as tissues where transgene expression is detected) after *in utero* gene delivery differs based on the route of administration. Numerous other studies have shown that different AAV serotypes have different tropisms in cell lines based on their surface prevalence of receptors. Zabner *et al* [41] demonstrated that AAV5 was more effective at binding to the apical surface of human airway epithelia and therefore results in better gene transfer as compared to AAV2. Although a few AAV serotypes have shown better transduction compared with the AAV2-based vectors, gene transfer efficiency in human airway epithelium has still not reached therapeutic levels because of pathophysiological barriers in human CF patients [42]–[44]. A major limitation for this is the lack of efficient vector transduction to airway epithelial cells through the apical surface. Significant progress is being made to achieve high-efficiency transduction of airway epithelium, including using chemicals and a combination of transduction-enhancing compounds after vector transduction [45]–[47], shortened AAV cassette [48], intramolecular joining of DNA or RNA from independent vectors [49]–[51], genetic modifications of the AAV capsids [52], and DNA shuffling combined with directed evolution [53]. Taken together with our findings, these studies suggest that AAV vector transduction efficiency and tropism are species specific, and by utilizing strategies such as vector pseudotyping and varying the time and route of vector administration, gene therapy efficiency can be improved. The human xenograft model may provide a very novel and promising tool for clinical/translational/drug efficacy research. In comparison with postnatal gene therapy, *in utero* gene therapy of airway epithelium and SMG for cystic fibrosis offers several potential advantages while posing unique challenges. The delivery of viral vectors directly to the trachea lumen would provide site-specific gene therapy, eliminating the need for systemic vector delivery and thereby decreasing the risks of germ-line transduction. Another advantage is the proximity of potential viral receptors on the respiratory epithelium and the submucosal glands in relation to the trachea lumen. In the pre-immune murine fetal environment, Bouchard *et al* [54] reported that AAV administration has the potential to induce tolerance and allow postnatal re-administration with a partially abrogated immune response. This response suggests that the animal has developed a partial immunologic tolerance to the virus. In previous reports, immunologic tolerance may be developed to the transgene, but not necessarily to the vector. This tolerance may have been due to the limited exposure in the pre-immune fetus to the vector. In contrast, the prolonged exposure to the expressed transgene stimulated a tolerogenic T-cell-mediated response [55], [56]. Another report by Boutin *et al* [57] reported that sero-prevalence of antibodies against AAV8 is only moderate in the human population when compared to AAV2, potentially facilitating immune escape of AAV8 vectors *in vivo*. This finding lends additional support to the use of a pseudotyping strategy for CFTR transgene delivery that allows for repeated administration using different capsid serotypes to maximize the efficiency of gene transfer and minimize immunologic reaction. Lastly, *in utero* CFTR replacement has the potential to correct the genetic defect before any manifestations of CF are present.

Despite the unprecedented efficiency of gene transfer observed with multiple vectors in our model, our results must be interpreted cautiously. The SCID mouse cannot mount an immune response, which may prolong transgene expression by the AAV vector. However, we did not observe a significant inflammatory reaction using AAV gene transfer in various models. Furthermore, the formation of membranes at both ends of the trachea provides an enclosed environment and allows prolonged exposure of the virus to the receptors of the fetal trachea. Although impossible to mimic in postnatal airways, this exposure is possible in the fetus because there is no physiologic gas exchange. Potential delivery of vector to the fetal trachea could be performed by endoscopic tracheal occlusion; this is currently being used in human clinical trials in Europe for congenital diaphragmatic hernia [58]. The human fetal trachea xenograft model can be used to screen vectors prior to this type of clinical application and the virus-receptor interaction can be studied in an environment representative of the developing human fetal trachea.

AAV-based vectors were utilized because of their long-term expression and low risk for insertional mutagenesis. However, Li *et al* [59] reported that AAV may randomly integrate at low frequency into mammalian chromosomes, preferentially integrating into transcribed genes in cells that are replicating. The near uniform transgene expression observed with AAV2/2 and AAV2/8 at 3 months after viral gene transfer suggests that integration events may have occurred and that integration into a respiratory stem cell population is likely. If the developing fetal tracheobronchial epithelium is more susceptible to insertional events than postnatal tracheobronchial epithelium, this observation has both positive and negative implications. If integration of AAV vectors occurs more readily in the fetal tracheobronchial stem cells, then correction of the CFTR mutation may be possible with in utero administration of a single site-specific AAV vector with a CFTR transgene. The human fetal trachea SCID xenograft model may be a useful means to study this phenomenon *in vivo* [60]. The downside of AAV integration is the potential of the AAV vector genome to influence chromosomal gene expression, either by inactivation of an adjacent gene, or more worrisome, by activation of an oncogene. Despite this concern, unlike retroviruses, there is recent evidence that demonstrates that AAV vectors are not associated with malignancy [59].

### Conclusions

We have shown highly efficient gene transfer by AAV pseudotyping in a validated model representative of human submucosal gland development. The stable expression of transgenes in a rapid cellular turnover environment, such as the trachea, suggests transduction of a stem cell population, possibly with insertional events, that can be easily studied in this model. *In utero* gene therapy for cystic fibrosis has considerable theoretical appeal. AAV vectors and the human fetal trachea SCID xenograft model can be effective tools for studying the mechanisms of efficient AAV transduction of stem cell populations, understanding AAV insertional events, and developing fetal gene therapy strategies for the treatment of cystic fibrosis.
